# Microalgae as novel drug-delivery system for biomedical field

**DOI:** 10.1080/10717544.2026.2616930

**Published:** 2026-01-27

**Authors:** Yueyou Dai, Dandan Guo, Aifang Li, Wei Chen, Yi Yang, Shuxuan Li, Lianhe Yang, Shuying Feng

**Affiliations:** a Medical College, Henan University of Chinese Medicine, Zhengzhou, People's Republic of China; b Henan Engineering Research Center for Chinese Medicine Foods for Special Medical Purpose, Zhengzhou, People's Republic of China

**Keywords:** Microalgae, delivery system, safety evaluation, targeted therapeutic, disease treatment

## Abstract

Currently, increasing attention is being paid to the extraction and utilization of materials with special biological activities in nature or for medical applications. Owing to their unique biological characteristics and diverse application potential, microalgae are among the most promising materials in the field of biomedicine. Because of their diverse morphology and readily functionalizable surface, they can efficiently carry drugs and achieve targeted drug release. This can avoid major challenges of other methods related to toxicity, biocompatibility, and immunogenicity, which is important for the treatment of various diseases, particularly those related to hypoxia. Despite the distinct advantages of microalgae over other biomaterials, several challenges persist in their practical application. Herein, we comprehensively describe the current state of research on the microalgae drug-delivery system (MDDS). In particular, we explore various microalgae-based strategies and methods to improve the load capacity and stability of DDS, and to achieve target positioning and tracking. With further research on microalgae, their application prospects in DDSs will broaden. In the future, researchers will continue to explore the features and advantages of microalgae; develop more efficient, safe, and accurate DDSs; and provide more options for clinical treatments. Continued progress in microalgal cultivation technology and reduction in large-scale production costs will expand the clinical applications of MDDSs.

## GRAPHICAL ABSTRACT

**Figure:**
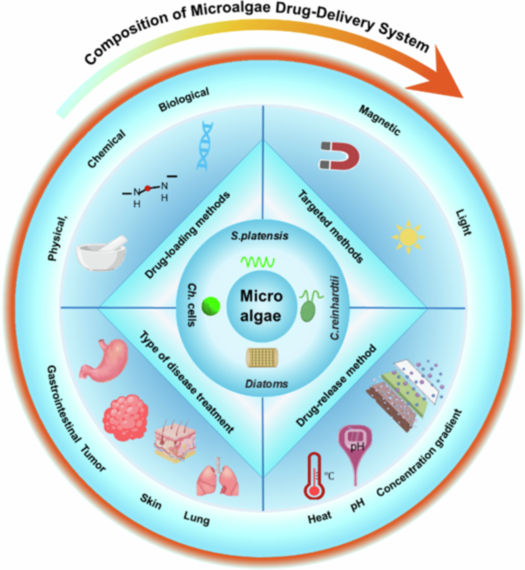

## 1 Introduction

The field of medicine depends on pharmacologically active agents, which are commonly known as drugs, to treat various diseases (Tibbitt et al. 2016). Despite a century of ongoing discovery and development, present-day drugs cannot be targeted *en masse* selectively at sites of interest. Drug molecules freely diffuse and are distributed throughout the body, leading to adverse events (Vincent et al. 2022; Bian et al. 2025). For example, owing to the lack of specificity of some agents, they act on various cells, destroying normal cells and leading to severe side effects and high toxicity (Qin et al. 2018; Li et al. 2023). Various cytotoxic agents can effectively treat diseases, and conventional cytotoxic therapies often eliminate diseases at the expense of damaging normal tissues, resulting in unacceptable toxicities. If an illness does not respond to an appropriate course of treatment, it is typically labelled as treatment-resistant (Howes et al. 2022). Drug resistance requires further high-dose drug administration, which often generates intolerable toxicity (Wang et al. 2024). Currently, to treat diseases, medications are commonly administered through various direct application methods, including inhalation (Ke et al. 2022), oral administration (Wang et al. 2021), injection (Chaudhary et al. 2019), and enema (Prasaja et al. 2022), which are facile and safe. However, low water solubility is a common trait of many therapeutic drugs that can impair their dissolution rate and oral bioavailability, presenting a difficult obstacle in the field of pharmaceutical development (Da Silva et al. 2020).

To overcome these negative effects, new disease treatment methods must be developed to enhance treatment efficacy, minimise side effects, and enable more precise therapies (Cao et al. 2025). Drug-delivery systems (DDSs) have been developed for effective drug delivery (Stefano 2023).

A DDS is a formulation or device designed to administer therapeutic substances, enhancing their efficacy and safety through controlled release at specific rates, times, and target sites within the body (Liu et al. 2024). Following the discovery that *β*-galactosidase-encapsulated red blood cells (RBCs) can treat Gaucher’s disease (Ihler et al. 1973), the utilisation of living cells as carriers in drug-delivery technology has attracted significant attention among researchers. This includes the use of RBCs, platelets, stem cells, leucocytes, and immunological cells (Yu et al. 2020). Compared with conventional DDSs, cell-based systems offer prolonged circulation, specific tissue tropism, superior flexibility, lower immunogenicity, and reduced cytotoxicity, all of which are attributed to their unique cellular properties. Cell-based DDSs possess intrinsic biodegradability and biocompatibility (Choi et al. 2023a). Despite demonstrating remarkable therapeutic benefits in preclinical studies, their clinical application faces several challenges, including the absence of straightforward and convenient methods for acquiring carrier cells and the lack of technologies to produce cytopharmaceuticals on a large scale while maintaining carrier-cell viability (Li et al. 2020).

Over the past few decades, microalgae, which are unicellular photosynthetic organisms, have attracted considerable attention in various fields ranging from biofuel production to medicine (Zhang et al. 2022c). They exhibit intriguing characteristics, such as rapid growth and high biomass efficiency, compared to plants (Alishah Aratboni et al. 2023). Owing to their active surfaces, microalgae can effectively adsorb functional molecules and metal elements, making them promising for DDSs. Microalgae are generally considered safe, and various administration methods have shown no significant toxicity (Wang et al. 2025). In addition, microalgae contain a variety of bioactive peptides with unique and diverse structures, which have a variety of biological activities, including antioxidant, antihypertensive, and antibacterial activities (Ashraf et al. 2025). Certain microalgae have proven suitable for drug delivery. For instance, *Spirulina platensis* (*S. platensis*) serves as a natural carrier for the construction of a drug-loaded system that facilitates targeted delivery and fluorescence imaging-guided chemotherapy for the treatment of lung metastasis in breast cancer. This system exhibits exceptionally high DLE% and pH-responsive sustained drug release (Zhong et al. 2020a). In addition, utilising *S. platensis* as a carrier to construct nanozyme-armed probiotic Lactobacillus plantarum (SP@LP@AuCe) system, and developed a micro-nanorobot that can be administered orally to improve the efficacy of oral probiotics and nanomedicine in the treatment of intestinal diseases (Hou et al. 2025). Surface-engineered *Chlorella* (Chl) modified with metal-organic framework (MOF) nanoparticles (ChL-MOF) can cooperate with photoacoustic dynamics and immunotherapy to alleviate tumour hypoxia. Under laser and ultrasound (US) irradiation, ChL-CHL generates reactive oxygen species (ROS), enhances photoacoustic effect and promotes tumour cell apoptosis (Xu et al. 2025).

In light of the shortcomings of the present cell-based DDSs, microalgae have emerged as promising candidates for optimising the effectiveness of drug delivery, giving rise to a microalgae drug-delivery system (MDDS). This paper presents the latest research on the application of microalgae in biomedicine. It provides an overview of the methods for loading drugs into microalgae, targeted therapeutic approaches, the detection of loaded drugs, safety analyses, and the application of microalgae in disease treatment. Furthermore, it outlines future applications of microalgae and their derivatives. Finally, current challenges and prospects of microalgal delivery are discussed. We expect that with more researchers engaged in microalgae-related studies, the application of microalgal materials in biomedicine will broaden, expanding their contributions to human health.

## 2 Advantages of MDDSs

**Figure 1.**
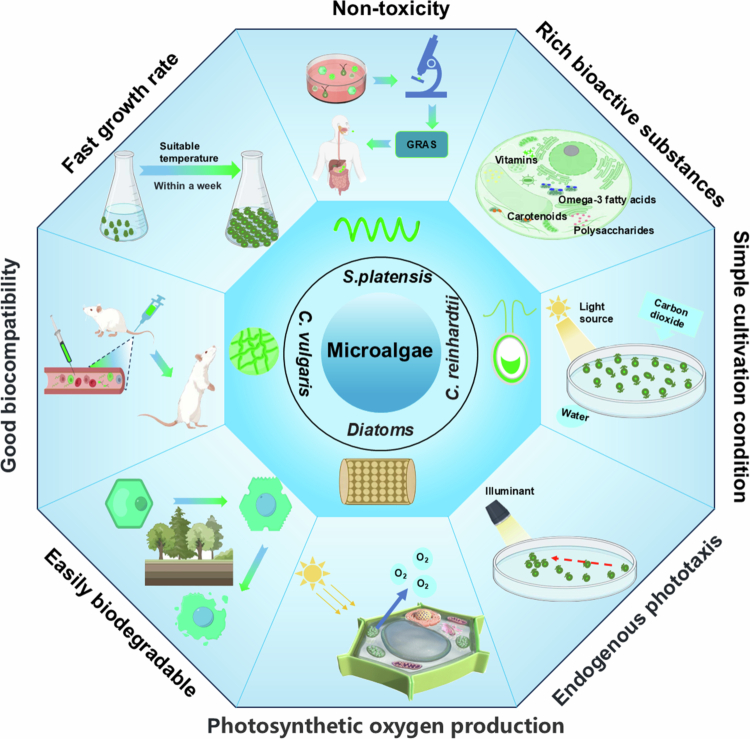
Advantages of microalgae-based drug-delivery systems. (The figure was created by the authors.).

Microalgae constitute a diverse assembly of single-cell photosynthetic organisms with a remarkable capacity for rapid growth under photoautotrophic conditions in various environments. Notably, they can be cultivated on nonarable land and can efficiently utilise saline and wastewater streams. Microalgae's broad environmental adaptability facilitates large - scale cultivation, offering a steady and ample raw material source for drug delivery systems, thereby guaranteeing their clinical stability and sustainability. (Bora et al. 2024; Gallego et al. 2025). Owing to their abundant bioactive compounds, distinctive surface architectures, ease of accessibility and cultivation, and exceptional biocompatibility, microalgae are highly promising DDSs in the biomedical field (Figure 1). First, their bioactive compounds include carotenoids, omega-3 fatty acids, polysaccharides, vitamins, and other bioactive chemicals. During drug delivery, these bioactive compounds boost the system's overall performance and may offer extra health perks to patients, enhancing treatment efficacy and their quality of life. (Kaur et al. 2025; Guil-Guerrero and Prates 2025). Second, in contrast to other microorganisms, viable microalgae typically exhibit minimal invasiveness toward mammalian cells or tissues, ensuring their superior biocompatibility, their robust safety profile, and the absence of genetic toxicity. This trait allows microalgae to serve as DDSs minimising harm to normal tissues, adverse effects, and immunogenicity, while enhancing the safety, reliability, and in - vivo drug delivery efficacy. (He et al. 2023; Jia et al. 2024). Third, microalgae demonstrate potent biological activities during oxygen-producing photosynthesis, making them invaluable for the management of oxygen-deficient conditions that exacerbate diseases.This natural characteristic stands as the exclusive advantage of microalgae in DDS. (Cohen et al. 2017; Chen et al. 2020; Li et al. 2024a). For instance, chlorophyll from *Spirulina* produces active oxygen when irradiated with a laser, which significantly enhances its phototoxicity toward tumour cells and accelerates tumour-cell apoptosis (Zhong et al. 2020b). Fourth, microalgal cells exhibit intrinsic phototaxis. Microalgae's phototaxis drives their active convergence on specific light sources, facilitating precise drug delivery, optimising treatment settings, and boosting DDS targeting and efficacy. (Verburg et al. 2021; Ferdewsi et al. 2025). Studies have demonstrated that red blood cell membrane-engineered algae (RBCM-algae) can successfully deliver oxygen *in situ* to tumour tissues under red-light-induced photosynthesis, thereby enhancing tissue oxygenation, alleviating tumour hypoxia, and ultimately improving radiotherapy outcomes (Qiao et al. 2020). Fifth, certain microalgal species, such as *S. platensis* (Abo et al. 2025; Zhong et al. 2020b) and *Chlamydomonas reinhardtii* (*C. reinhardtii*) (Wakabayashi et al. 2021), possess unique motility characteristics that allow them to actively migrate to targeted disease niduses. Such active motility empowers microalgal to precisely target drug delivery at lesion sites, elevating local drug levels, amplifying therapeutic outcomes, and minimising normal tissue drug exposure and side effects. For instance, a new type of biocompatible biohybrid microswimming device was developed that utilises single-cell freshwater green algae *C. reinhardtii* as the driving source. Experimental results indicated that this device can maintain cell compatibility when co-cultured with normal and tumour cells. Moreover, certain microalgae are employed as DDSs because of their distinctive characteristics. For example, diatom frustules consist of biosilica self-assembled into intricate porous shells. These shells exhibit large specific surface area, biocompatibility, customisable surface chemistry, thermal stability, and remarkable mechanical and chemical resistance (Min et al. 2024).

## 3 Construction strategies for MDDSs

### 3.1 Physical adsorption

**Figure 2.**
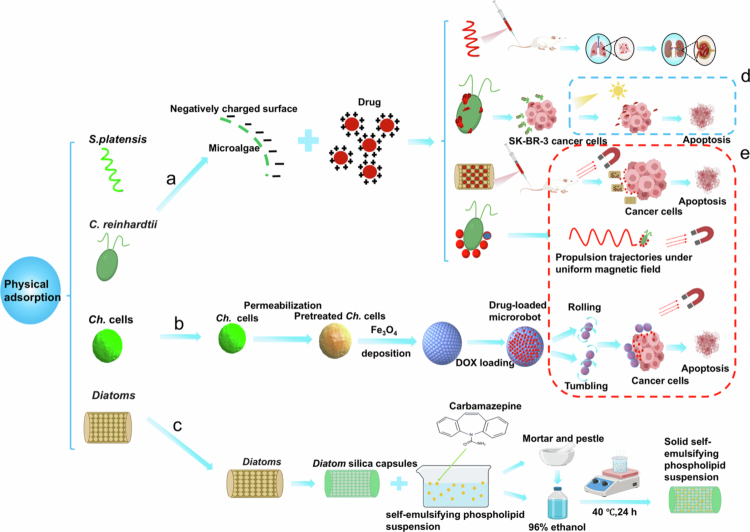
Representative examples of physical integration. a) DDS is formed by electrostatic adsorption between the drug and the microalgae; b) to improve the efficiency of drug binding, the cell membrane of the algae is modified by using charged magnetic Fe_3_O_4_ nanoparticles; c) through grinding and solvent-assisted methods, the algae are made to be efficient for drug loading; d) the phototaxis of microalgae itself can be used to make them gather at the site of action via light guidance, for improving the therapeutic effect; e) combination with magnetic nanoparticles gives microalgae magnetic properties, allowing them to be guided using an external magnetic field to improve the therapeutic effect. (The figure was created by the authors.).

Physical adsorption involves incubating drug carriers with a concentrated solution of the selected drug. In many cases, medications bind to surfaces and are later discharged via diffusion driven by concentration gradients (Figure 2) (Vona et al. 2023). Utilising a physical method, the MDDS exhibits favourable stability and biological activity during drug loading. For instance, a self-emulsifying drug-delivery system (SEDDS) consisting of octyl polyethylene glycol-8 glyceryl esters, phospholipids, propanediol, caprylic acid, and capric acid triglycerides served as a solid carrier for an oral DDS. In this context, diatomite (*DE*) was prepared using two distinct methods. The initial technique involved carefully integrating *DE* with a stable dispersion of carbamazepine (CBZ) in self-emulsifying drug-delivery systems to create solid self-emulsifying phospholipid suspensions (SSEPSs). Alternatively, *DE* was dispersed in an ethanol solution containing CBZ and SEDDS components, followed by evaporation of the ethanol. Notably, after 10 weeks of accelerated storage conditions (40 °C and 70% relative humidity), the crystallinity and solubility of CBZ in the SSEPSs remained largely unchanged, whereas an increase in crystallinity was observed in solid dispersions. This underscores the efficacy of *DE* for adsorbing liquid CBZ in SEPSs, which preserves its initial properties (Milović et al. 2014). Moreover, a biologically inspired design strategy entails the noncovalent assembly of a thin, flexible, and uniform coating around active microorganisms. This strategy, leveraging polymeric nanoparticles as synthetic materials and *Rhinomonas*—a unicellular green algae—as a biological model, achieved a coating assembly efficiency of approximately 90%. Chitosan—a natural biopolymer—is used to encapsulate microalgal cell walls. This flexible surface coating not only preserves the viability and phototaxis capabilities of microalgae but also allows further engineering to accommodate the requirements of biomedical cargo molecules. Consequently, a proof-of-concept biomedical application was demonstrated in which nanoparticles embedded within a thin coating, coupled with the chemotherapeutic agent doxorubicin (DOX), facilitated controlled drug delivery to tumour cells (Akolpoglu et al. 2020).

Magnetic nanorobots have emerged as promising platforms for targeted drug delivery, and numerous strategies have been developed in recent years. DDSs can load drugs through physical adsorption and enhance their targeting ability via magnetic-field guidance. However, achieving precise and efficient drug delivery *in vivo* remains a challenge, with biocompatibility and multifunctional integration posing significant obstacles (Zhang et al. 2024). To address these issues, researchers have investigated and developed delivery systems for microalgae. For example, small molecules can be co-loaded with iron oxide nanoparticles on diatoms (Kang et al. 2024). With the application of an external magnetic field, the diatoms, once administered systemically, can be directed toward tumours. This research underscores the vast potential of diatoms as innovative and powerful therapeutic delivery systems. Magnetic biohybrid microrobot multimers (BMMs) developed using *Chlorella* (*Ch.*) have also demonstrated promise for targeted drug delivery. Initially, *Ch*. cells (approximately 3–5 μm in diameter) were magnetised with Fe_3_O_4_ to load DOX. Through attraction-induced self-assembly and magnetic-dipole interactions, these cells were reversibly converted into chain-like BMMs, such as dimers and trimers. Under a magnetic field of 70 Gs, the BMMs could swim at a speed of 107.6 μm/s, exhibiting excellent manoeuvrability. The results indicated that the BMMs possessed low cytotoxicity, along with a high DOX-loading capacity, and achieved pH-triggered drug release. These attributes make BMMs effective for targeted drug delivery in cancer treatments (Gong et al. 2022). In addition, by utilising the unicellular freshwater green microalga *C. reinhardtii* as a power source, a biocompatible hybrid microswimmer was developed. This microswimmer incorporates magnetic spheres functionalized with polyelectrolytes (1 µm in diameter), which are noncovalently attached to the microalgae surface and enable three-dimensional (3D) swimming. In tests under various physiologically relevant conditions, the microswimmer exhibited good cytocompatibility when co-cultured with both healthy and cancerous cells. Moreover, by using biohybrid microalgae as an active transport vehicle, fluorescent isothiocyanate-dextran molecules can be efficiently delivered to mammalian cells. The enhanced biocompatibility and motility of this newly designed biohybrid microswimmer make it a promising candidate for targeted drug delivery in medical applications (Verburg et al. 2021). However, mass fabrication of microrobots with precise propulsion and therapeutic efficacy remains challenging. Researchers proposed a facile technique using *S. platensis* as a biotemplate to mass-produce magnetic microrobots with multiple functions. In *S. platensis* cells, core–shell Pd@Au nanoparticles were synthesised as photothermal conversion agents, and Fe_3_O_4_ nanoparticles were deposited on their surfaces to enable magnetic actuation. The microrobots were also loaded with DOX to enhance their chemotherapeutic effect. In a rotating magnetic field, the biohybrid microrobots exhibited efficient propulsion capability, with a maximum speed of 526.2 μm/s, and excellent chemical–photothermal therapy effects. In addition, robots can be separated when exposed to near-infra-red (NIR) irradiation from a laser, triggering pH- and NIR-controlled drug release, which makes them promising for a wide range of applications in drug loading, targeted delivery, and chemical–photothermal therapy (Wang et al. 2019). New strategies are essential to effectively treat bacterial infections, reduce the emergence of drug-resistant strains, and minimise side effects, particularly in skin and soft-tissue infections.

### 3.2 Chemical modification

To address the limitations associated with direct drug administration, synthetic nanoparticles, including porous silicon nanoparticles, have been widely used for drug delivery. However, the synthesis of these nanoparticles (NPs) presents hurdles related to the scalability, cost, and environmental impact of potentially toxic materials. Materials derived from natural resources have been explored (Kang et al. 2024). For instance, a surface-initiated atom transfer radical polymerisation technique was employed to graft methacrylate oligomer (ethylene glycol) copolymers onto diatom biosilica microcapsules. The resulting composites exhibited remarkable thermal responsiveness, making them potential candidates for drug-delivery applications (Vasani et al. 2015). Another study investigated the impact of engineered surface chemistry on the drug loading and release characteristics of *DE* particles. Various silane compounds, such as (3-aminopropyl)triethoxysilane (APTES), mPEG-silane, octadecyltrichlorosilane (OTS), and tris (hydroxymethyl) aminomethane, have been utilised to convert diatoms into mesoporous degradable silicon nanoparticles (SiNPs). SiNPs are characterised by a high surface-to-volume ratio, intense fluorescence, a substantial drug encapsulation capacity, biodegradability, and the absence of cytotoxicity. When loaded with anticancer drugs, they demonstrated sustained drug release at various pH values and enhanced *in vitro* cytotoxicity. Given their low cost and superior attributes, SiNPs derived from abundant natural materials are promising alternatives to synthetic nanoparticles (Maher et al. 2016). In addition, *DE* is a promising carrier for synthetic silica-based materials in drug-delivery applications. Engineered surface chemistry plays a pivotal role in the drug-loading and release properties of *DE* particles. Four silanes (APTES, mPEG-silanes, OTS, and (3-glycidyloxypropyl)trimethoxysilane (GPTMS)) and two phosphonic acids (2-carboxyethylphosphoryl acid (2-CEPA) and 16-phosphonohexadecanoic acid (16-PHA)) were employed for surface modification to enhance the loading and release of water-insoluble drugs (such as indomethacin (IND)) and water-soluble drugs (such as gentamicin). Hydrophilic *DE* surfaces containing carboxyl, amine, or hydrolysed epoxy groups extended the release of IND, whereas hydrophobic *DE* surfaces containing organic hydrocarbons prolonged the release of gentamicin. By customising the drug-loading and discharge behaviour of small *DE* particles, this study revealed their potential for the delivery of both hydrophobic and hydrophilic drugs (Bariana et al. 2013a). A novel method was proposed to harness the energy generated by intact cells as a biological motor. In this context, the unicellular biflagellate algae *C. reinhardtii* serve as ‘microoxen.’ This approach involves attaching loads (polystyrene beads with diameters ranging from 1 to 6 µm) to the cells using surface chemistry, controlling their movement through phototaxis, and employing photochemistry to release the loads. These motile microorganisms are capable of transporting microscale loads (3-µm-diameter beads) at speeds of approximately 100-200 μm/s, covering distances of up to 20 cm (Weibel et al. 2005).

Magnetic microrobots are promising for biomedical applications such as targeted delivery and therapy. Researchers developed an innovative DDS tailored to address skin disorders by leveraging the benign green alga *C. reinhardtii*. In this system, vancomycin undergoes chemical modifications to bind to *C. reinhardtii*. Upon activation by UVA1 light, the photocleavable linkers release antibiotics, effectively halting the growth of *Bacillus subtilis* (*B. subtilis*). This study demonstrated the potential of utilising living organisms as drug-delivery vehicles, where UVA1 irradiation controls antibiotic release, opening new possibilities for therapeutic applications (Shchelik et al. 2020). Notably, *C. reinhardtii* has emerged as an exemplary drug carrier owing to its biodegradability, established safety (Generally Recognised As Safe, GRAS), non-immunogenicity (Schenck et al. 2015), inherent antibiotic production capacity (Bhowmick et al. 2020), and amenability to further genetic engineering (Stoffels et al. 2017). This live drug carrier demonstrated remarkable success in inhibiting the proliferation of *B. subtilis*. The use of a living organism as a DDS for controlled antibiotic release through UVA1 irradiation was demonstrated, indicating potential for future advancements (Gao et al. 2019; Zhang et al. 2020).

The adoption of chemically loaded DDSs utilising microalgae presents a viable solution to address the limitations associated with conventional disease treatment methodologies. Specifically, sonodynamic therapy (SDT) (Liang et al. 2020)—a noninvasive technique employed for localised antitumor treatment—faces hurdles such as inadequate tumour accumulation of SDT agents, hypoxic conditions within tumours, and protective autophagy mechanisms. To overcome these challenges, researchers have devised targeted DDSs that employ surface-engineered microalgae such as *Ch*. to supply oxygen sustainably through photosynthesis (Masojídek et al. 2021). *Macrophage-mimetic Ch.* (*mchl*) is generated by encapsulating *Ch.* within the membrane of macrophages, enhancing their compatibility with biological systems and enabling tumour-targeted aggregation through the inflammatory homing effect inherent to the macrophage membrane (Oroojalian et al. 2021). Furthermore, the integration of *β*-cyclodextrin (*β*-CD) into the membrane coating yields CD-modified *mchl* (CD-mchl), which can accommodate lipid insertions. It exploits the host–guest interactions between CD-mchl and liposomes that have been modified with adenosine deaminase (ADA-NP) (Van Tran et al. 2019). Supramolecular complexes of mchl-NPs were constructed. These complexes enable the simultaneous transport of *Ch.*, hematoporphyrin, and chloroquine phosphate (encapsulated within the ADA-NPs) to cancerous tissues. By integrating localised oxygenation, SDT, and autophagy suppression, this combined strategy significantly improved the effectiveness of mchl-CQ-HP-NP in combating melanoma. Tumour rechallenge experiments revealed that these interventions induced favourable alterations in the tumour microenvironment, including alleviation of hypoxia, induction of immunogenic cell death through SDT, and inhibition of autophagy. These changes collectively elicit potent antitumor immune responses and memory (Gao et al. 2023).

**Figure 3.**
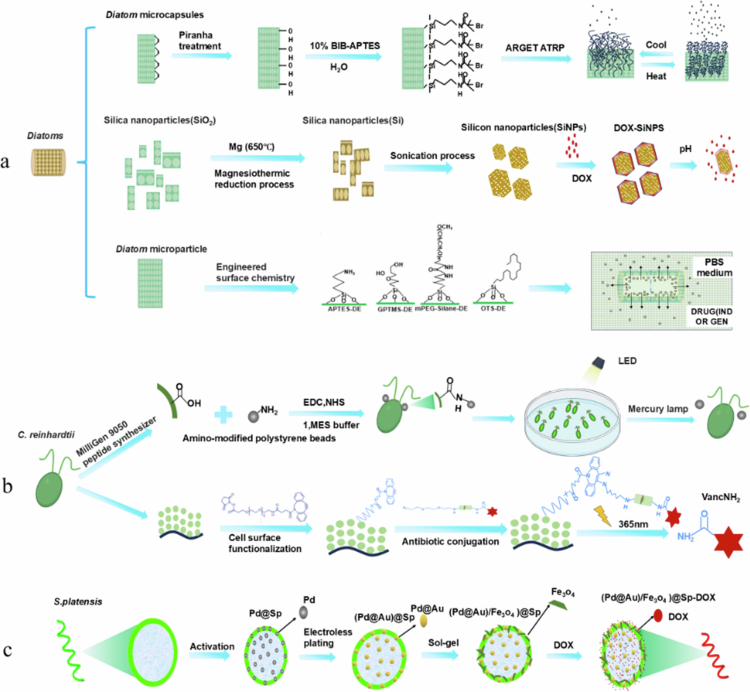
Chemical modification strategies for the preparation of microalgae. a) *Diatoms* can be modified via three strategies: (1) Surface chemical groups exposed post-treatment, bond with BIB-APTES via cold-hot changes for drug loading/release; (2) Magnesium-thermal redox reaction exposes pore structure for pH-controlled drug loading/release; (3) Surface covalently bonds with groups like APTES-DE, releasing drugs via concentration gradients. b) After treatment, the chemical groups can be exposed on the cell surface of *C. reinhardtii*, which can be modified in two ways: (1) Cell surface hydroxyl and drug surface amino groups form bond via dehydration-condensation, fixing drug for phototaxis-guided delivery; (2) Algae surface covalently bonds with drug surface; photosensitive groups in bonds release drug under 365-nm irradiation. c) *S. platensis* binds to Pd metal ions and then Fe3O4, forming a DDS for easier drug loading and magnetic field-enhanced targeting. (The figure was created by the authors.).

Microalgae can be engineered into specialised drug carriers through various chemical modifications, including loading of chemical groups, membrane encapsulation, and incorporation of magnetic particles (Figure 3). Surface engineering and chemical alteration of loaded chemical groups can optimise drug loading capacity and release profiles. Membrane encapsulation technology has attracted attention for *in vivo* drug delivery because of its exceptional biodegradability and non-immunogenicity. The magnetic-drive approach precisely guides the microalgae to the target position. Compared with physically modified microalgae, chemically modified microalgae exhibit enhanced stability and can achieve precise targeting and intelligent drug release, thus offering greater versatility and efficacy in DDSs. However, these processes are relatively intricate, and the safety of the utilised materials must be meticulously ensured to prevent the elicitation of an immune response.

### 3.3 Bioreactor

**Figure 4.**
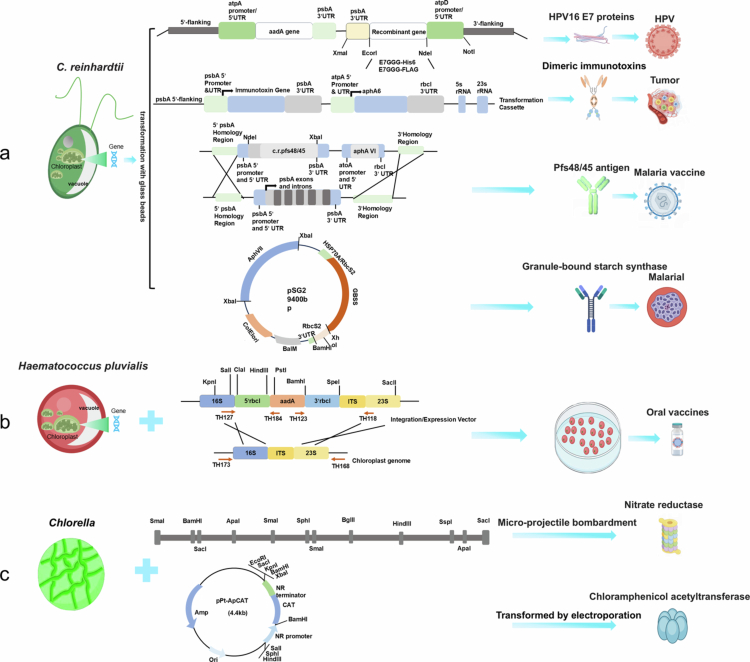
Biological reaction strategies for the preparation of microalgae. a) *C. reinhardtii*: By modifying the chloroplast genes with different gene fragments, corresponding proteins can be synthesised, such as HPV16 E7 proteins for treating HPV, dimeric immunotoxins for tumour prevention and treatment, and antibodies related to malaria treatment. b) *H. pluvialis*: By modifying its chloroplast genes, it can be used to produce oral vaccines. c) *Ch*: The use of different gene fragments obtained via genetic modification allows the synthesis of nitrate reductase and chlorocatechol acetyltransferase. (The figure was created by the authors.).

The basic principle of a bioreactor is to utilise the biological functions of organisms (such as microorganisms) or enzymes to simulate the environment inside an organism *in vitro* and perform biochemical reactions (He et al. 2024). Compared with existing bioreactor systems, microalgae represent a highly appealing alternative for the production of pharmaceuticals, recombinant proteins, and other valuable products (Figure 4). Various genetically engineered microalgal species, such as *C. reinhardtii* (Scarfe et al. 2025), *Chlorella vulgaris* (*C. vulgaris*) (Lacurezeanu and Vodnar 2025), *Haematococcus pluvialis* (*H. pluvialis*) (Alateyah et al. 2022), and *Phaeodactylum tricornutum* (*P. tricornutum*) (Wang et al. 2025), have been used to synthesise diverse products, including vaccines, antibodies, and enzymes (Feng et al. 2023; Scarfe et al. 2025). Research on the biological responses of the chloroplast system has been conducted using *C. reinhardtii* and *H. pluvialis*, whereas studies on *C. vulgaris* have primarily focused on the nuclear system. The chloroplast system of *C. reinhardtii* is an excellent platform for the expression of complex recombinant proteins, highlighting its immense potential as a bioreactor. Studies on *C. reinhardtii* have largely focused on the biological response of the chloroplast system, whereas studies on *C. vulgaris* have primarily focused on the nuclear systems. Meanwhile, the chloroplast system of *C. reinhardtii* is particularly well-suited for the expression of complex recombinant proteins, highlighting its potential as a bioreactor.

By functioning as an exemplary system for the expression of the E7GGG protein in a soluble and immunogenic format, the chloroplast system in *C. reinhardtii* emphasises the potential advantages of microalgae for vaccine production in controlled and sterile conditions (Scarfe et al. 2025). Researchers have demonstrated the generation and deposition of monomeric and dimeric immunotoxins in the algal chloroplasts. These proteins are formed by the fusion of an antibody region recognising CD22-a B-cell surface antigen—fused to the functional domain of *Pseudomonas aeruginosa* exotoxin A, which possesses enzymatic activity. Studies have indicated that immunotoxins produced by algae accumulate as active, soluble proteins that can bind to target B cells and efficiently kill them *in vitro*. Additionally, the administration of either monomeric or dimeric immunotoxins significantly enhanced the survival of mice with human B-cell tumours (Tran et al. 2013). Another study explored the use of *C. reinhardtii* chloroplasts to produce intricate recombinant polypeptides. This study successfully demonstrated that the C-terminal antigenic portion of Pfs 48/45 could be expressed in these chloroplasts, resulting in a recombinant protein that, owing to its precise conformation, was detectable by existing transmission-blocking antibodies (Jones et al. 2013). Researchers have identified and characterised endogenous chloroplast sequences as efficient genetic tools for transforming *H. pluvialis* chloroplasts using microprojectile bombardment. These findings expand the potential of *H. pluvialis* as a platform for expressing recombinant proteins in various biotechnological applications, such as the development of oral vaccines for aquaculture (Gutiérrez et al. 2012). Furthermore, by employing microprojectile bombardment, the nitrate reductase (NR) gene from *C. vulgaris* was successful introduced into NR-deficient mutants of *Ch. sorokiniana*. The resulting stable transformants could grow on a nitrate-based medium, and this growth capability was retained even after numerous passages in both selective and nonselective settings. These transformants exhibited inducible NR activity matching that of the wild-type cells (Dawson et al. 1997).

Microalgae are currently used as bioreactors in various applications, including disease treatment and food processing. For example, *Thalassiosira pseudonana*—a diatom—has been genetically modified to display the IgG-binding domain of protein G on a biosilica surface. This allows the attachment of cell-targeting antibodies, resulting in the selective targeting and killing of neuroblastoma and B-lymphoma cells when conjugated with drug-loaded nanoparticles. Additionally, treatment with biosilica led to tumour growth regression in a subcutaneous mouse xenograft model of neuroblastoma (Delalat et al. 2015). Moreover, researchers have optimised the transformation system of *C. vulgaris* by utilising the promoter and gene terminator for NR sourced from *P. tricornutum* (Niu et al. 2011). Related studies have examined the development of genetically engineered starch granules derived from the single-celled green alga *C. reinhardtii*, which contains candidate antigens for malarial parasites (Dauvillée et al. 2010). Notably, owing to their advanced protein manufacturing apparatus and ability to undergo post-synthetic modifications, microalgae have emerged as a green and sustainable option for the manufacture of pharmaceutical glycoproteins. Meanwhile, certain microalgae, e.g. *C. reinhardtii, Dunaliella bardawil,* and species of *Ch.*, have been approved as safe for human consumption by the U.S. Food and Drug Administration (FDA). The rigid cell walls of microalgal cells can naturally encapsulate recombinant proteins, protecting them from harsh stomach environments. This characteristic enables the rapid manufacturing and targeted administration of S-glycoprotein and soluble ACE2, which could contribute to the treatment and suppression of SARS-CoV-2 (Dehghani et al. 2022). In conclusion, microalgae have a wide range of potential applications as bioreactors, and their biological synthesis capabilities make them suitable as delivery vehicles for protein-based therapeutic drugs.

## 4 Evaluation of MDDS stability and controllability

### 4.1 Identification of surface features following drug loading

In the study of MDDS, in order to determine whether the drug is successfully loaded and evaluate the effectiveness of the loading process, a variety of detection techniques have been widely used, including morphological structure observation, direct observation of the loading condition, surface properties detection, particle size analysis, and chemical composition analysis. Scanning electron microscopy (SEM) is an important tool for morphological observation. For example, when studying mesoporous silica (DE), field emission SEM (Quanta 450) showed that the pore morphology of silane and phosphonic acid modified DE did not change, but most of the pores closed after drug introduction. It was confirmed that the drug was adsorbed on the DE surface with multiple layers and filled the pores (Bariana et al. 2013a). The cross-section of the sample can be observed by transmission electron microscopy (TEM). For example, nanoparticles (Pd@Au NPs) composed of gold (Au) and palladium (Pd) with a JEOL JEM-1400 have a core-shell nanostructure, indicating that the prepared BMMRs are suitable for drug loading. Moreover, the average particle size of Pd@Au NPs on *Spirulatus* obtuse cells was 14.79 ± 4.05 nm (Wang et al. 2019). Direct observation of loading using fluorescence imaging technology, using fluorescent labelled drug or microalgae fluorescence signal, by examining the signal intensity and distribution to determine the drug absorption and loading, such as SP@DOX in *spirulina* cells to produce red fluorescence, The presence of DOX in SP was confirmed by light field microscopy and UV-vis (UV-vis) spectroscopy (Zhong et al. 2020a), and SEM imaging and microscopic experiments also provided evidence for the attachment of labelled antibiotics to the surface of *Chlamydiae rheinarum* (Shchelik et al. 2020; Gao et al. 2023). Surface properties detection is achieved by zeta potential measurement, which can understand the surface charge characteristics of the drug-loaded material, such as the negative charge of the microrobots and the positive charge of DOX, and BMMRs can load DOX through electrostatic interaction (Wang et al. 2019). The successful loading of the drug was verified by the zeta potential changes and FT-IR analysis after the adsorption of DOX by silicon nanoparticles (SiNPs) (Maher et al. 2016), and the reduction of zeta potential caused by the loading of positively charged DOX onto SP (Zhong et al. 2020a). Particle size analysis is critical to determine the particle size distribution of microalgae. For example, laser light scattering is used to verify the accuracy of purified DE particle size (Bariana et al. 2013a), and dynamic light scattering is used to determine the stability of ADA-NP after storage, indicating its excellent stability (Gao et al. 2023). Chemical composition analysis can reveal the surface functional groups and potential chemical modifications of microalgae, such as the characterisation and measurement of BMMRs by field emission scanning electron microscopy equipped with energy dispersion spectrometer, X-ray diffraction, and inductively coupled plasma mass spectrometry (ICP-MS) (Wang et al. 2019). The chemical composition and functional groups of DE drugs before and after incorporation were analysed by energy dispersive X-ray spectroscopy and Nicolet 6700 FT-IR spectrometer (Bariana et al. 2013a). In summary, the combined application of these techniques can comprehensively and accurately determine whether the drug is successfully loaded in microalgae, and provide a strong basis for evaluating the effectiveness of the drug loading process.

### 4.2 Evaluation index of drug-loading success rate and encapsulation rate

The DLE% of microalgae, which serve as DDSs, is assessed using the following method. First, the amount of DOX in the supernatant was quantified using a standard concentration curve generated from a series of DOX solutions of varying concentrations and measured at an absorption wavelength of 480 nm. Using this equation, the DLE% of BMMRs was quantified. The BMMRs’ drug-release performance was assessed under two pH conditions: pH 7.4 and pH 5.4. The solution was heated via NIR laser irradiation for 30 min, followed by a 30-min interval during which no irradiation was applied. Second, the SP@DOX product was obtained via centrifugation, and a positive association was established between the colour of the culture medium supernatant and the starting concentration of the drug. The DLE% and encapsulation efficiency (EE%) were calculated using the UV-vis and fluorescence spectra of the culture supernatants, along with a standard curve of DOX solutions. The DLE% of SP exceeded 85% for all tested DOX concentrations, and compared with prior studies on micro/nanocarriers, the drug encapsulation efficiency was significantly higher (Zhu et al. 2010; Yang et al. 2013; Alapan et al. 2018). Researchers have investigated the effects of diverse drug-loading periods on *S. platensis* samples. From 2 to 12 h, the intensity of the red signals and the absorbance of the residual DOX decreased consistently. However, after 24 and 48 h, a green colour and absorption peaks indicative of *S. platensis* appeared, indicating structural disruption. The encapsulation efficiency increased with the drug-loading time until it reached a maximum at 12 h; further extension caused an efficiency reduction owing to *S. platensis* structural damage, indicating that 12 h is the optimal drug-loading time (Zhong et al. 2020a).

### 4.3 Evaluation of drug-release performance and pharmacokinetics

An optimal DDS for biomedical applications requires controlled drug release that aligns with physiological responses and clinical demands. Microalgae-based DDSs are influenced by several factors that affect drug-release performance, including the pH of the delivery region, surface hydrophilicity and hydrophobicity of the carrier, drug distribution within the carrier, and concentration gradients of the drug. The interplay among these factors determines the drug’s release rate and efficacy during delivery. The pH of the delivery region plays a pivotal role in regulating drug release because various drugs exhibit differing sensitivities to pH changes. Using a spectrophotometer (UV-3600, Shimadzu), researchers found that adjusting the pH of the delivery region could modify the solubility of the drug and consequently influence its release behaviour (Wang et al. 2019). For instance, studies on pharmaceuticals of DOX from SiNPs at two distinct pH values (5.5 and 7.4) indicated varying release capabilities, as analysed using ICP-MS. The cumulative drug-release graphs revealed two distinct phases: burst release and slow sustained release from the porous SiNPs, highlighting the pH dependence of the release kinetics. During the first 6 h, greater burst release of DOX was observed at the lower pH (5.5), with 32% of the drug being released, compared to 22% at pH 7.4. This was likely due to the rapid desorption of the drug from the surface of the silicon microspheres (Maher et al. 2016). The hydrophilicity and hydrophobicity of the carrier surface also significantly affect drug adsorption and release. By modifying the carrier surface hydrophobicity or introducing specific functional groups, drug–carrier interactions can be regulated, influencing the adsorption and release performance. Researchers have reported the release kinetics of IND—a non-aqueous soluble drug encapsulated in *DE* microparticles with different surface properties. The drug displayed two-phase release behaviour, exhibiting a rapid onset of release during the initial 6 h, followed by sustained and slow release over a period ranging from 13 to 26 d (Souza et al. 2009). Functionalization with APTES, GPTMS, and 2-CEPA reduced the burst release for IND from unaltered *DE* may rise by as much as 20%. This indicates that a hydrophilic surface improves drug adsorption and encapsulation in *DE* pores and matrices, leading to slower release. Thermogravimetric analysis was performed to determine the drug loading, and UV spectrophotometry was used to analyse *in vitro* drug release from the modified *DE* microparticles. The results indicated that hydrophilic surfaces containing polar carboxyl, amine, or hydrolysed epoxy groups facilitated the sustained release of IND. In contrast, the sustained-release profile of gentamicin on hydrophobic *DE* surfaces that had been modified by the addition of organic hydrocarbons was enhanced (Bariana et al. 2013a). Notably, optimal drug distribution enables controlled release, while concentration gradients enhance drug penetration and diffusion, both of which affect the release performance. The potential use of diatom silica in implants and oral drug delivery was demonstrated using IND, which achieved a high drug loading of 22 wt.% and maintained sustained release over a two-week period, as evidenced by curve-fitting methods. Drug release from diatom structures exhibited a rapid surface deposition phase, followed by slow, sustained release via diatom pores and the internal structure following zero-order kinetics. These findings support the use of diatom silica as a natural biocarrier for drug delivery and a replacement for synthetic silica materials (Aw et al. 2012). Furthermore, the density- and ratio-dependent confrontational effects of the two drugs in dual DDSs for resistant tumours represent a major obstacle, as they may impair the efficacy of individual therapeutic agents and promote the proliferation of tumour cells. To address this issue, a novel layered composite embedded with *DE* and capable of housing two chemotherapeutic drugs in isolated chambers of a single carrier was developed, and drug release was measured using high-performance liquid chromatography and UV spectrophotometry. This strategy allows the precise release of compounds while maintaining the ideal molar composition, which is essential for achieving synergistic results under *in vitro* conditions (Kabir et al. 2020). In the design and optimisation of microalgae-based DDSs, it is necessary to comprehensively consider and adjust these factors to achieve stable and efficient drug delivery. Precise drug delivery can be achieved by controlling the pH of the delivery region, adjusting the surface properties of the carrier, and optimising the drug distribution and concentration gradients, leading to improved therapeutic outcomes.

### 4.4 Safety evaluation after drug loading

Nanoparticles and microparticles provide a multitude of benefits when used as DDSs. However, their interactions with cellular, tissue, and organ components within living organisms have raised concerns regarding their potential negative effects. These interactions may lead to alterations and disruptions at the cellular level, compromising an individual’s post-treatment well-being. To evaluate the cytotoxicity of the microalgae-based DDSs, two commonly used methods were employed: 3-(4,5-dimethylthiazol-2-yl)-2,5-diphenyltetrazolium bromide (MTT) and fluorescence-based assays. Using two cell lines—RAW 264.7 macrophages and MDA-MB 231-TXSA—researchers initially examined the toxicity of SiNPs. For both cell lines, no notable poisonous effects were observed across the tested doses (from 10 to 250 μg/mL) after a 24-h incubation period (Park et al. 2009; Peng et al. 2014; Maher et al. 2016). *In vitro* experiments demonstrated that *DE* nanoparticles exhibited minimal toxicity to cells, even at concentrations as high as 300 μg/mL and exposure times of 72 h. The findings suggested that *DE* nanoparticles can serve as safe nanocarriers (Rea et al. 2014). The viability of chemical–photothermal therapy cells was assessed using the MTT assay and live/dead staining. 769-*P* cells were exposed to BMMRs reporters at concentrations ranging from 0 to 2000 μg/mL. All samples exhibited cell viabilities exceeding 80% even at elevated concentrations, indicating minimal cytotoxic impact (Wang et al. 2019). Additionally, the biomedical compatibility and adverse effects of both bare and modified *DE* nanoparticles were determined by examining the adenosine triphosphate (ATP) activity in MCF-7 and MDA-MB-231 cells that had been exposed to them (Santos et al. 2010). The ATP concentration was quantitatively determined using the celltiter-Glo® luminescent cell viability assay. MCF-7 and MDA-MB 231 cells exposed to increasing concentrations of *DE* nanoparticles for 24 h exhibited minimal toxicity (Terracciano et al. 2015). These findings further support the potential of *DE* nanoparticles as safe and effective DDSs.

Currently, the safety assessment concerning other nanoparticles utilised as drug delivery vehicles has encompassed several pivotal aspects, namely long - term biological compatibility, biodegradable properties, immune - related reactions, behaviours within living organisms, and organ - targeted toxicity.

When it comes to long - term biological compatibility, prolonged interactions between diverse nanomaterials and biological tissues and cells can give rise to a variety of scenarios. These include the possible liberation of detrimental substances (Tseriotis et al. 2025) and the emergence of chronic inflammatory responses (Bakkar et al. 2025). Such events have the potential to disrupt the normal physiological state of organisms; Regarding biodegradable properties, the degradation routes, speeds, and by - products of nanomaterials within living organisms are intricately linked to their safety profile (Idrees et al. 2020). Different types of nanomaterials may produce degradation products that inflict damage on organisms. For instance, the build - up of specific small - molecule substances in the body can initiate toxic reactions (Zheng et al. 2025); In the context of immune - related reactions, the interplay between nanoparticles and the immune system involves multiple facets, such as endotoxemia, complement activation, and the ability to provoke an immune response (Liu et al. 2022). When nanoparticles enter an organism as foreign entities, they can trigger immune reactions. Moreover, over - activation of the immune system may lead to adverse outcomes, including inflammatory and allergic reactions (Toscano and Torres-Arias 2023); As for behaviours within living organisms, imaging technologies and radioactive labelling methods, among others, are employed to monitor the dynamic alterations of nanoparticles. This enables the determination of their accumulation in various tissues and organs, as well as their metabolic and excretion pathways. Based on these in - vivo behaviours, it is possible to ascertain whether nanoparticles pose potential toxicity (Garrigós et al. 2024); Concerning organ - targeted toxicity, certain nanoparticles exhibit specific toxic effects on particular organs. Different types of nanoparticles have distinct toxic mechanisms when acting on vital organs like the liver, kidneys, heart, and lungs (Dantas et al. 2022; Shang et al. 2024). Through systematic toxicological tests, the functional and structural changes in organs after exposure to nanoparticles can be detected, which holds significant reference value for ensuring the safety of nanomedicines in clinical settings. For example, iron oxide nanoparticles have demonstrated potential toxic impacts on organ systems including the nervous system, heart, and lungs (Chrishtop et al. 2021). Given the immense application potential of microalgae in the realm of drug delivery systems, it is imperative to draw on the well - established safety evaluation methods for other nanoparticles. This will facilitate more in - depth and comprehensive research to guarantee the safe utilisation of microalgae.

## 5 Applications of MDDSs

MDDSs play a crucial role in improving drug targeting, enhancing therapeutic efficacy, and maintaining microalgal vitality. Numerous studies have confirmed that microalgae can significantly enhance the therapeutic effects for many diseases, including gastrointestinal diseases, tumours, lung diseases, skin diseases, and diabetes. This section provides an in-depth analysis of the application scope, design principles, and therapeutic effects of microalgae, detailing the current progress and future development directions of microalgal DDSs in the field of disease treatment (Table 1).

**Table 1.** Application of MDDS in disease treatment.

Types of microalgae	Disease	Type(s) of drug	Functional characteristics	References
*C. reinhardtii*	*Pseudomonas eruginosa* pneumonia	Antibiotic	Quickly and evenly distributed into deep lung tissue, with low clearance rate and long retention time in the trachea	(Zhang et al. 2022c)
	Gastrointestinal diseases	DOX	Rapid and persistent swimming behaviour prolongs local retention in the gastrointestinal tract	(Zhang et al. 2022b)
	HeLa cells	Fluorescent sothiocyanate-dextran	Biocompatibility and mobility	(Yasa et al. 2018a)
	Human breast cancer	Addition of terbium	Capable of carrying large loads, magnetically guided directional movement, non-cytotoxic, imaging tracking	
	SK-BR-3 cancer	Doxorubicin	Strong adhesion to drugs and phototactic targeting properties	(Akolpoglu et al. 2020)
	Skin disease	Vancomycin	Safe release of drugs through light irradiation	(Shchelik et al. 2020)
	Wound	Oxygen, antibiotic	Immediate release of oxygen during illumination, high biocompatibility	(Kolackova et al. 2020)
	Wound	Vascular growth promoting factor	Enhances the ability of endothelial cell tube formation	(Jarquín-Cordero et al. 2020)
	Chronic wounds in diabetes	Heparin	Oxygen is used to alleviate hypoxia and improve delivery efficiency to deep wound sites	(Choi et al. 2023b)
*Diatom*	J774 osteoclasts-like	Sodium alendronate	Good bone conduction ability and activation of tissue regeneration	(Cicco et al. 2019)
	Uncontrolled haemorrhage	Chitosan	Good biocompatibility, ideal haemostatic effect	(Feng et al. 2016)
	Colorectal cancer	Vitamin B12, cisplatin, 5-fluorouracil	Long retention time, strong adhesion, easy release	(Delasoie et al. 2018)
	Colon cancer	Mesalamine and prednisone	Maintains drug release and enhances drug permeability	(Zhang et al. 2013)
	Skin disease	Naproxen	Low cost, large surface area, mesoporous properties, and biocompatibility	(Vona et al. 2023)
*S. platensis*	Pathogenic bacterial infection	Polydopamine	Robust propulsion, natural fluorescence, tailored biodegradation, and selective cytotoxicity	(Xie et al. 2020)
	Radiation-induced injury	Astaxanthin	Improves drug solubility, gastric stability, cellular uptake, and intestinal absorption	(Zhang et al. 2023)
	Chronic Periodontitis	Antioxidant	Strong antioxidant properties	(Kaipa et al. 2022)
	Diabetic wounds	Chitosan	Accelerates wound healing, reduce pro-inflammatory cytokines IL-1 *β* and TNF-*α*, and increase the quantity of anti-inflammatory cytokine IL-10	(Hendrijantini et al. 2020)
	Skin disease	Lecithin-bile salt-integrated	High stability, site-specific drug delivery, controlled and sustained drug release, and fewer side effects	(Zewail et al. 2022)
	Radiation-induced intestinal damage	Amifostine	Comprehensive drug accumulation and effective radiation protection benefit gut microbiota homoeostasis and long-term safety	(Zhang et al. 2022a)
*Chlorella Vulgaris*	Diabetic wounds	Hydrogen	Selectively reduces highly toxic •OH and ONOO species and reduces inflammation and has excellent biocompatibility and ROS scavenging features	(Chen et al. 2022)
	Diabetic foot ulcers	Nitric oxide and oxygen	Accelerates wound closure, re-epithelialization, and angiogenesis and increases the survival rate of skin grafts	(Chen et al. 2023)
	Diabetic wounds	Polyacrylamide and ALG	Excellent skin adhesion, photosynthesis, production of oxygen and electrons, improvement of hypoxic conditions, and promotion of cell proliferation	(Wu et al. 2024)
	Diabetic bacterial infected wounds	Poly(ionic liquid)-based microneedles	Relieves deep wound hypoxia through local targeted disinfection	(Davani et al. 2022)
	Diabetic wounds	Oxygen	Relieves local hypoxia, increases angiogenesis, and promotes extracellular-matrix synthesis to significantly accelerate wound closure	(Wang et al. 2022)
	Lead poisoning	Berberine and carboxymethyl chitosan/ALG	Helps retain lead in the gastrointestinal tract for a longer period of time, effectively removing lead from the liver, kidneys, and bones	(Liu et al. 2023a)
	4T1 breast cancer cells	Oxygen	Increases local oxygen levels and resensitizes drug-resistant cancer cells to radiotherapy and phototherapy	(Qiao et al. 2020)
	Diabetes	Insulin, ALG	A potent and sustained hypoglycaemia effect, coupled with favourable biodegradability and biosafety profiles.	(Ren et al. 2023)
*Synechococcus elongatus*	Cardiovascular disease	Hydrogel microparticles	Transports oxygen to ischaemic tissue through photosynthesis	(Stapleton et al. 2023)
	Chronic wounds in diabetes	Dissolved oxygen	Enhances wound oxygenation, fibroblast proliferation, and angiogenesis; nontoxic and non-immune responsive	(Chen et al. 2020)
*Haematococcus*	Chronic wounds in diabetes	Astaxanthin	Eliminates bacteria, supplies oxygen, eliminates ROS, promotes angiogenesis, and significantly accelerates wound healing	(Kang et al. 2024)
*microalgae*	Chronic wounds in diabetes	Dissolved oxygen	Promotes angiogenesis and proliferation, inhibits inflammatory response in chronic wounds, releases dissolved oxygen to alleviate wound hypoxia	(Jin et al. 2024)
	Wound	Oxygen, recombinant human growth factors	Strong mechanical stress and resistance to freezing	(Centeno-Cerdas et al. 2018)

### 5.1 Gastrointestinal diseases

When used to treat gastrointestinal diseases, microalgae-based DDSs exhibit robust drug aggregation and provide significant radiation shielding, surpassing the efficacy of free drugs and enteric-release capsules. For example, researchers have utilised *S. platensis* as a microcarrier of amifostine to develop an oral delivery system. This system demonstrated superior drug accumulation and effective radioprotection throughout the small intestine compared to free drugs and their enteric capsules. It prevents radiation-induced intestinal injury, prolongs survival, and does not affect tumour regression. Additionally, it enhances the stability of gut microbiota, and its long-term safety has been confirmed (Zhang et al. 2022a). Researchers developed an algae-based exercise platform that utilises the swimming behaviour of microalgae to extend the gastrointestinal retention time. This platform embeds pH-sensitive capsules to enhance small-intestine delivery. Compared to magnesium-based micromotors, algal motors significantly improve dye distribution and enhance chemotherapy load retention. By combining natural algal motors with capsule protection, a promising micromotor platform was developed to improve gastrointestinal cargo transport for biomedical applications (Zhang et al. 2022b). In addition, researchers developed SP@asxnano—an oral microalgae-based nanocomposite system aimed at addressing the challenges posed by radiation-induced injuries to both the intestinal tract and the entire body. This system combines the natural microalga *S. platensis* with astaxanthin nanoparticles (asxnano). *S. platensis* cooperates with asxnano to enhance drug delivery, improving its distribution in the intestine and bloodstream. *S. platensis* minimises drug loss in the stomach, prolongs intestinal retention, and ensures constant release and gradual degradation of asxnano. Asxnano enhances drug solubility, cellular uptake, and intestinal absorption. The collaboration between *S. platensis* and asxnano has various functions, including anti-inflammation, microbiota protection, and upregulation of faecal short-chain fatty acids. Moreover, this system ensures long-term biosafety during drug administration. By combining the special attributes of microalgae with nanoparticles, this system expands the range of medical applications of *SP*, positioning it as a highly adaptable DDS (Zhang et al. 2023).

### 5.2 Tumours

By combining the special attributes of microalgae with nanoparticles, this system extends the medical reach of SP, positioning it as an exceptionally adaptable DDS (Kotulová et al. 2025). Researchers developed a strategy to address tumour hypoxia by harnessing a natural photosynthetic system based on engineered *C. vulgaris* to produce oxygen (O_2_) directly within the tumour site. To optimise the delivery of *C. vulgaris* to tumour tissues while minimising macrophage ingestion and systemic elimination, researchers introduced an innovative technique for engineering the RBC membrane to function as a surface modifier for algae (Mohandas and Gallagher 2008; Hu et al. 2011; Fang et al. 2018). The resultant RBCM-algae are efficiently transported to tumour tissues by utilising red-light-induced photosynthesis to generate O_2_, enhancing tissue oxygenation and mitigating tumour hypoxia. This augments the efficacy of radiation therapy. Furthermore, laser-irradiation-induced chlorophyll release from microalgae generates reactive oxygen species (ROS) for photodynamic therapy, further enhancing cancer-cell eradication (Qiao et al. 2020). Conventional anticancer drugs have drawbacks including poor water solubility and poor pharmacokinetics, which hinder chemotherapy. These limitations contribute to severe adverse side effects and multidrug resistance in patients (Marchal et al. 2015). Researchers have synthesised a new biomaterial composed of marine microalgae *DE* particles and vitamin B12 for the targeted delivery of anticancer drugs to colorectal cancer. This material can be loaded with multiple drugs, among which ruthenium complexes have unique release characteristics, remain in the material for up to 5 d in aqueous media, and are easily released in lipophilic environments, such as cell membranes. The B12 coating increases cell adhesion and facilitates targeted delivery. These results indicate that the developed biomaterial can effectively deliver water-insoluble drugs to tumours (Delasoie et al. 2018).

In cancer therapy, poor targeting of therapeutics, which results in severe adverse effects on normal tissues, is a significant obstacle (Liu et al. 2024a). Researchers developed a biohybrid algal microswimmer system with a high manufacturing yield, achieved through the molecular assembly of the nonliving component around the cell wall. The nonliving component, composed of a conformal layer of the natural biopolymer chitosan, is electrostatically attached to the negatively charged cell wall of *C. reinhardtii*. As a binding agent, chitosan significantly enhances the adhesion of drug carriers. Importantly, the thin chitosan nanoparticle coating does not affect the motility and phototactic properties of the biohybrid microalgae. Researchers have demonstrated the potential of these biohybrid microalgae by modifying the attached nanoparticles with a photocleavable linker and using them to selectively deliver chemotherapeutic cargo (DOX) to SK-BR-3 cancer cells. This high-throughput manufacturing technique for biohybrid microswimmers lays the groundwork for the evolution of next-generation microalgae-based cargo delivery platforms based on microalgae (Akolpoglu et al. 2020). Porous silica extracted from diatoms exhibits considerable potential for medical applications owing to its low cost, simple purification process, environmental characteristics, and unique features. Studies have indicated that diatom particles transport mesalazine and prednisone with low toxicity, enhance drug permeability, and improve drug bioavailability. These findings suggest that PDMS is a non-cytotoxic biomaterial with potential as a drug carrier (Zhang et al. 2013).

### 5.3 Pulmonary diseases

Microalgae have unique advantages as drug mediators for the treatment of lung diseases. Researchers employed click chemistry to create a hybrid microrobot that efficiently delivers antibiotics to the lungs using nanoparticles wrapped in neutrophil membranes and microalgae. The robot runs at a high speed in simulated lung fluid, ensuring a uniform distribution of drugs and a long tissue retention time. In a mouse model of pneumonia, it significantly reduced the bacterial load, reduced the mortality rate, and exhibited low toxicity. Research has indicated that algae–nanoparticle hybrid robots have considerable potential in critical care, particularly in lung treatment (Zhang et al. 2022c).

### 5.4 Skin diseases

Microalgae are the preferred carriers for treating skin diseases because of their biocompatibility and rich nutritional value. Researchers have validated the efficacy of the *n*-octyl chain as a skin DDS through *in vitro* experiments and 3D tissue simulations of drug penetration. The octyl chain enhances the adhesion of the skin to *DE*, achieving stable drug release and penetration with good biocompatibility, indicating its broad application potential in the treatment of skin diseases (Vona et al. 2023). Concurrently, researchers developed a new DDS for the targeted treatment of skin diseases using the green alga *C. reinhardtii*. After chemical modification, vancomycin binds to a photolytic linker and is masked by Reinhardt algae. Studies have indicated that bacterial growth is only inhibited under UVA1 illumination (Shchelik et al. 2020). *S. platensis* (SPR) is a nutrient-rich blue-green alga with antioxidant, immunomodulatory, and anti-inflammatory effects. To improve skin drug delivery, researchers developed an *S. platensis*-loaded bilosome (SPR-BS) DDS. This system is safe, efficient, and capable of continuously releasing drugs. Evaluation after UVB irradiation confirmed its photoprotective and anti-aging effects. Compared with SPR alone, SPR-BS has better anti-aging effects and is effective for treating UV-induced skin damage (Zewail et al. 2022).

A promising strategy for wound healing involves the use of photosynthetic biomaterials, specifically a wound dressing composed of a photosynthetic alginate hydrogel that incorporates *C. reinhardtii* microalgae. These hydrogels can release additional bioactive molecules, including recombinant VEGF or antibiotics, and research has indicated that these dressings, which can be customised, are effective in promoting wound healing (Corrales-Orovio et al. 2023). Researchers have created photosynthetic sutures that can locally release oxygen and growth factors, while providing mechanical fixation. *In vitro* experiments confirmed that oxygen and growth factors are continuously released and that sutures are stable under mechanical stress and freezing conditions. This indicates that the development of novel bioactive sutures using photosynthetic gene therapy has considerable potential to advance regenerative medicine (Centeno-Cerdas et al. 2018). Furthermore, receptor phosphorylation and *in vitro* angiogenesis assays were performed to assess the biological activity and functionality of the recombinant pro-angiogenic growth factors produced by algae. The results demonstrated that combining transgenic strains expressing any of the three growth factors had a synergistic effect on the ability of endothelial cells to form tubes. These findings support the application of transgenic algae expressing pro-angiogenic growth factors in wound-healing strategies (Jarquín-Cordero et al. 2020).

### 5.5 Diabetes

Recently, using *C. vulgaris* crosslinked with sodium alginate (ALG), a microalgae-based oral insulin delivery system (*C. vulgaris*@INS@ALG) was developed. *C. vulgaris*@INS@ALG overcomes the gastrointestinal barrier, protects insulin from gastric conditions, and achieves pH-responsive drug release in the intestine. It facilitates insulin absorption through its direct release from the delivery system and endocytosis by M cells and macrophages. In a streptozotocin -induced type 1 diabetic mouse model, *C. vulgaris*@INS@ALG demonstrated long-lasting hypoglycaemia effects compared to direct insulin injection without causing intestinal tract damage. Furthermore, long-term oral administration of the carrier *C. vulgaris*@ALG improved gut microbiota dysbiosis and increased probiotic *Akkermansia* abundance in db/db type 2 diabetic mice, enhancing insulin sensitivity. Microalgal insulin delivery systems are biodegradable and biosafe after oral administration. This strategy provides a natural, efficient, and multifunctional alternative for oral insulin delivery (Ren et al. 2023).

## 6 Discussion

Microalgae have demonstrated significant accomplishments in the realm of drug delivery, yet they continue to grapple with the issue of low drug delivery efficiency (Zhou et al. 2023a). Moreover, microalgae, when employed as drug carriers, confront a plethora of challenges and constraints. Firstly, strain variability poses a considerable hurdle. Different strains of microalgae exhibit variations in their genetic, physiological, and biochemical characteristics. These differences can lead to substantial disparities in drug loading capacity and release profiles when utilised as drug carriers, thereby influencing the stability and consistency of drug delivery systems (Laurens et al. 2014). Scalability also presents a major obstacle. Although some progress has been made in microalgae culture technologies, numerous technical and cost-related barriers persist in achieving large-scale, industrial production to meet the demands of drug delivery systems. For instance, ensuring the stability of the microalgae growth environment during large-scale cultivation and effectively controlling production costs remain pressing issues (Chen et al. 2018). Biocompatibility and safety are also non-negligible factors. As foreign substances entering the human body, microalgae may trigger immune reactions or other adverse effects, and their long-term safety requires further investigation (Wang et al. 2025). In terms of storage stability, microalgal carriers may undergo structural or functional alterations during storage, potentially impacting the preservation and subsequent delivery efficacy of drugs (Kang et al. 2026). Furthermore, the current regulatory policies and standards for microalgae as drug carriers are imperfect, which to some extent restricts their clinical application promotion (Deniz et al. 2025). Hence, constructing efficient Drug Delivery Systems (DDSs) is of paramount importance.

When microalgae are harnessed as bioreactors for antibody drug production, enhancing protein expression levels becomes imperative. One promising approach to achieving this goal is through the application of CRISPR base-editing technology (Dhokane et al. 2020). The processing of algal biomass and other algal products involves multiple steps, including cultivation, harvesting, and extraction. Harvesting, which entails extracting concentrated algal slurry from the growth medium, accounts for 20%–30% of production costs and poses both technical and economic challenges (Mirakhorli et al. 2023). For example, efficiently separating microalgae from the medium during harvesting without damaging the cells and reducing energy consumption and reagent costs are urgent problems to be addressed (Nguyen et al. 2024).

When utilising microalgae as a drug delivery system, multiple factors must be taken into account, encompassing the characteristics of both the drug and the algae. These considerations facilitate the selection of an appropriate delivery method to ensure timely and precise drug release. The initial step is to evaluate whether a drug necessitates a delivery system based on its unique properties. For instance, tannins such as epigallocatechin gallate (EGCG), gallic acid, and ellagic acid present challenges for pharmaceutical applications due to their poor lipid solubility, low bioavailability, unpleasant taste, and short half-life in humans (Cai et al. 2017). To overcome these issues, suitable drug carriers are required to enhance their applicability. Osmotic-pump drug-delivery systems utilise pressure to achieve controlled release of the active ingredient with minimal influence from the gastrointestinal environment. Drug release is influenced by factors such as solubility, osmotic pressure, pore size, and the controlled release membrane. Modulating these factors can lead to the development of osmotic systems suitable for preprogrammed drug delivery, which are flexible and versatile for both systemic and targeted drug delivery. Additionally, selecting a delivery route tailored to the location and physiology of the target site is crucial. For example, topical delivery holds greater potential than systemic delivery for treating local and certain systemic diseases. Moreover, injecting a drug-polymer combination into a specific site to form a semi-solid drug reservoir offers the advantages of simple operation and local continuous drug delivery. Consequently, numerous in situ polymer delivery systems have been developed and extensively studied for delivering various drugs. For drugs that require direct application to the skin or mucosa, topical delivery methods such as gels (Yao et al. 2023), lotions (Li et al. 2022), and patches (Zhou et al. 2023b) can be employed. For oral or injectable delivery to specific body sites, appropriate dosage forms such as capsules (Zhang et al. 2022b), tablets (Liu et al. 2023b), solutions, or injections (Kim et al. 2023) can be utilised. The selection of an appropriate microalgal vector is pivotal in determining the delivery route. Microalgae with modified cell walls to encapsulate drugs (Guo et al. 2017) or microalgae extracts can serve as drug carriers (Zhao et al. 2020). Finally, designing an appropriate delivery system necessitates selecting drug encapsulation and release methods based on previous studies on drug loading methods. By considering these factors, microalga-based drug delivery systems can be customised to meet specific therapeutic needs and ensure effective and precise drug delivery.

Genetic engineering holds immense potential in shaping future research directions. The precise modification of microalgae through gene editing technology is anticipated to further enhance their performance as drug carriers. For instance, attempts can be made to edit genes related to the cell wall of microalgae to optimise the cell wall structure and improve drug loading capacity (Tang et al. 2024). Alternatively, regulating genes involved in metabolic pathways related to drug release can achieve more precise drug release control (Wang et al. 2025). Improving drug loading is also a significant research direction. Researchers can explore novel drug loading strategies, such as developing innovative chemical conjugation methods to attach drugs to the surface or inside microalgae more efficiently (Khavari et al. 2021). Alternatively, utilising the biosynthetic pathway of microalgae to directly synthesise drugs within microalgae cells can enhance drug loading (Dehghani et al. 2022). The development of hybrid systems is also noteworthy. Combining microalgae with other materials, such as polymers and nanoparticles, to construct hybrid drug delivery systems can leverage their respective advantages and achieve superior drug delivery effects (Li et al. 2024b). Moreover, numerous gaps remain to be addressed before clinical translation, including in-depth studies on the behaviour and fate of microalgal carriers in complex organisms, such as their distribution in vivo, metabolic pathways, and clearance mechanisms (Ye et al. 2025). Large-scale pre-clinical studies should be conducted to evaluate the safety and efficacy of microalgal drug delivery systems to provide a solid foundation for clinical application (Bo 2025).

In recent years, microalgae have been employed as bioreactors to produce antibody drugs and can be combined with gene editing technology to enhance protein expression levels. For example, gene editing technologies such as zinc-finger nucleases, transcription activator-like effector nucleases, and CRISPR-Cas nuclease systems have been utilised to boost the production of microalgal metabolites. Among these methods, the CRISPR-Cas system is the most efficient and widely adopted in genome editing, encompassing transformation methods, Cas9 and single-stranded guide RNA (sgRNA) expression strategies, gene knock-in/knockout methods, and CRISPR interference expression modification (Zhang et al. 2019). CRISPR-Cas systems have revolutionised the field of genetic modification by providing a simple and precise method applicable at the genomic level. Despite the limited availability of genomic resources, remarkable progress has been achieved in both genetic and metabolic aspects of microalgae engineering. These genomic resources have been used as 'safe harbour' sites to achieve stable transgene expression in microalgae (Jeong et al. 2023). Additionally, the CRISPR system is increasingly being used for precise genome engineering and endogenous genome regulation in microalgae research (Banerjee et al. 2018). Current research focuses on the therapeutic efficacy of alga-derived metal nanoparticles against microbial infections and cancer (Khalid 2020). Furthermore, genomics and proteomics are powerful tools for elucidating the molecular mechanisms triggered by nanoparticle exposure. Using a variety of physiological, biochemical, and molecular approaches, researchers have analysed the effects of cadmium selenide/zinc sulphide quantum dot (CdSe/ZnS quantum dot, QD) exposure on the marine diatom *P*. tricornutum. The results showed that adaptation to QDs could alleviate their inhibitory effect on the growth of Phaeodinium trigonum cultures. Increased glutathione levels in the lag phase indicate that the cells are under stress. Studies of the transcriptional expression of certain stress response genes revealed upregulation of these genes in algae exposed to quantum dots. Comparative proteomic analysis of exposed and unexposed cells revealed numerous differentially expressed proteins (Morelli et al. 2015). Moreover, to expand the applications of diatom nanotechnology beyond biosilica production, it is necessary to develop strategies to isolate diatom shells and alter their chemical composition. By modifying, integrating, transforming, or mimicking diatom biosilica shells using chemical or biological methods while preserving their structural features, scalable and cost-effective methods can be developed for producing a variety of nanostructured smart materials (Ragni et al. 2018).

Advances in microalgae cultivation techniques have enhanced the growth efficiency, yield, and quality of microalgae and promoted their applications in energy, food, and pharmaceutical fields. These advances include improved cultivation systems, photosynthesis, carbon fixation metabolism, harvesting techniques, and the use of novel bioreactors. Ultra-high throughput algal harvesting has been achieved using microfluidic rigid spiral microchannels (Mirakhorli et al. 2023). Strategies have been developed to enhance light-driven reducing power generation and optimise CO2 assimilation (Naduthodi et al. 2021). A scalable and environmentally friendly hydrothermal process has been developed to maximise the utilisation of microalgal biomass. Carbon quantum dots derived from microalgae have been developed for high-contrast bioimaging. Self-flocculation is a low-cost, environmentally friendly, and safe method. However, further systematic studies are needed to explore the long-term safety and dose-related immunocompatibility of xenobiotics before their clinical application. In terms of research limitations, most current research on microalgae as drug carriers is still in the laboratory stage, and there is a long way to go before large-scale clinical application. Moreover, existing studies on the stability and performance changes of microalgal carriers in different physiological environments are not in-depth enough, and there is a lack of long-term follow-up research data (Malik et al. 2020). In the future, it is essential to strengthen multidisciplinary cooperation and integrate knowledge from biology, materials science, medicine, and other fields to jointly overcome the challenges faced by microalgae drug delivery systems. Simultaneously, it is necessary to enhance communication and cooperation with regulatory authorities, promote the formulation and improvement of relevant regulatory policies and standards, and provide a guarantee for the clinical transformation of microalgal drug delivery systems (He et al. 2025). Nevertheless, microalgae have demonstrated immense potential in biomedical applications. As more researchers dedicate themselves to microalgae-related research, future studies are expected to expand the application of this unique material in the field of drug delivery.

## Data Availability

We agree to make data and materials supporting the results or analyses presented in their paper available upon reasonable request.
